# Orphan nuclear receptor Nur77 affects cardiomyocyte calcium homeostasis and adverse cardiac remodelling

**DOI:** 10.1038/srep15404

**Published:** 2015-10-21

**Authors:** Lejla Medzikovic, Cees A. Schumacher, Arie O. Verkerk, Elza D. van Deel, Rianne Wolswinkel, Ingeborg van der Made, Natascha Bleeker, Daniella Cakici, Maarten M. G. van den Hoogenhof, Farid Meggouh, Esther E. Creemers, Carol Ann Remme, Antonius Baartscheer, Robbert J. de Winter, Carlie J. M. de Vries, E. Karin Arkenbout, Vivian de Waard

**Affiliations:** 1 Department of Medical Biochemistry, University of Amsterdam, The Netherlands; 2 Department of Cardiology, University of Amsterdam, The Netherlands; 3 Department of Clinical and Experimental Cardiology, University of Amsterdam, The Netherlands; 4 Department of Anatomy, Embryology and Physiology, Academic Medical Center, University of Amsterdam, The Netherlands; 5 Division of Cardiology, Thoraxcenter, Erasmus Medical Center, University of Rotterdam, The Netherlands; 6 Department of Cardiology, Tergooi Hospital, Blaricum, The Netherlands

## Abstract

Distinct stressors may induce heart failure. As compensation, β-adrenergic stimulation enhances myocardial contractility by elevating cardiomyocyte intracellular Ca^2+^ ([Ca^2+^]_i_). However, chronic β-adrenergic stimulation promotes adverse cardiac remodelling. Cardiac expression of nuclear receptor Nur77 is enhanced by β-adrenergic stimulation, but its role in cardiac remodelling is still unclear. We show high and rapid Nur77 upregulation in cardiomyocytes stimulated with β-adrenergic agonist isoproterenol. Nur77 knockdown in culture resulted in hypertrophic cardiomyocytes. Ventricular cardiomyocytes from Nur77-deficient (Nur77-KO) mice exhibited elevated diastolic and systolic [Ca^2+^]_i_ and prolonged action potentials compared to wild type (WT). *In vivo*, these differences resulted in larger cardiomyocytes, increased expression of hypertrophic genes, and more cardiac fibrosis in Nur77-KO mice upon chronic isoproterenol stimulation. In line with the observed elevated [Ca^2+^]_i_, Ca^2+^-activated phosphatase calcineurin was more active in Nur77-KO mice compared to WT. In contrast, after cardiac pressure overload by aortic constriction, Nur77-KO mice exhibited attenuated remodelling compared to WT. Concluding, Nur77-deficiency results in significantly altered cardiac Ca^2+^ homeostasis and distinct remodelling outcome depending on the type of insult. Detailed knowledge on the role of Nur77 in maintaining cardiomyocyte Ca^2+^ homeostasis and the dual role Nur77 plays in cardiac remodelling will aid in developing personalized therapies against heart failure.

Myocardial contractility and heart rate are increased in response to stimulation of cardiomyocyte β-adrenergic receptors by catecholamines, to enhance cardiac function on demand^1^. Different cardiac insults, such as cardiomyopathies, myocardial infarction and loss of contractile function due to elevated pressure, also lead to β-adrenergic receptor activation, which acts as a compensatory mechanism to maintain cardiac output^1^,^2^. If these insults persist however, chronic overstimulation of β-adrenergic receptors occurs, enhancing maladaptive myocardial remodelling, involving hypertrophy, fibrosis and cardiomyocyte death, eventually impairing contractility and heart failure. The deleterious potency of β-adrenergic overstimulation is demonstrated by improved clinical outcome after inhibition of β-adrenergic receptor activation by β-blockers, currently the first-line therapy for heart failure^3^. With a prevalence of more than 23 million heart failure patients worldwide, and 5- and 10-year survival rates of respectively 50% and 10%, heart failure is one of the leading causes of death in the Western world^4^. Hence, unravelling the molecular pathophysiology in the early stages of cardiac remodelling, before the onset of functional heart failure, is of great importance for the development of novel therapeutic strategies.

Activation of β-adrenergic Gs-protein-coupled receptors induces activation of signalling cascades, leading to larger intracellular calcium ([Ca^2+^]_i_) transients, necessary for enhanced contractility. Larger increases in [Ca^2+^]_i_ will lead to stronger contractions^5^. In addition, [Ca^2+^]_i_ homeostasis is essential to regulate excitation-contraction coupling, maintain electrophysiological balance, and control transcriptional regulation by activating key enzymes such as Ca^2+^-calmodulin-activated phosphatase calcineurin^6^. Consequently, altered cardiomyocyte Ca^2+^ homeostasis is an important characteristic of diseased myocardium and plays a central role in the pathophysiology of cardiac remodelling and heart failure^7^.

Orphan nuclear receptor Nur77, also known as NR4A1, NGFI-B or TR3, is an immediate-early gene which is involved in cellular stress responses, metabolism, proliferation, differentiation and apoptosis in various cell types. In vascular disease, we and others have shown that Nur77 inhibits smooth muscle proliferation^8^,^9^, macrophage accumulation^10^,^11^, pro-inflammatory macrophage polarization^10^,^11^,^12^,^13^, lipid loading/foam-cell formation^13^, and matrix metalloproteinase production^9^. Together with the promotion of a contractile phenotype in smooth muscle cells^8^,^9^, enhanced endothelial cell survival and endothelial cell-driven angiogenesis^14^, Nur77 has been attributed an atheroprotective role in the arterial vessel wall. At present, the role of Nur77 in the heart is less clear. Nur77 has been shown to mediate cardiomyocyte apoptosis upon oxidative stress^15^ and in response to angiotensin II-induced pressure overload, Nur77-deficient (Nur77-KO) mice and rats with cardiac-specific Nur77 knock-down exhibit attenuated adverse cardiac remodelling^16^. However, after myocardial infarction, Nur77-KO mice were shown to present worsened outcome^17^. Together these reports imply a dual role for Nur77 in cardiac disease. Interestingly, β-adrenergic stimulation has been shown to significantly up-regulate Nur77 expression in the heart^18^ implying a role for Nur77 in β-adrenergic overstimulation-induced cardiac remodelling. Very recently, it was shown that Nur77 overexpression in murine hearts could attenuate hypertrophy after β-adrenergic overstimulation^19^. Here, we assess the effect of Nur77 deficiency on calcium homeostasis in cardiomyocytes, in cardiac remodelling induced by chronic β-adrenergic stimulation, and in relation to cardiac pressure overload by transverse aortic constriction (TAC).

## Results

### Nur77 expression in the healthy heart and isoproterenol-challenged cardiomyocytes

**Figure 1:**
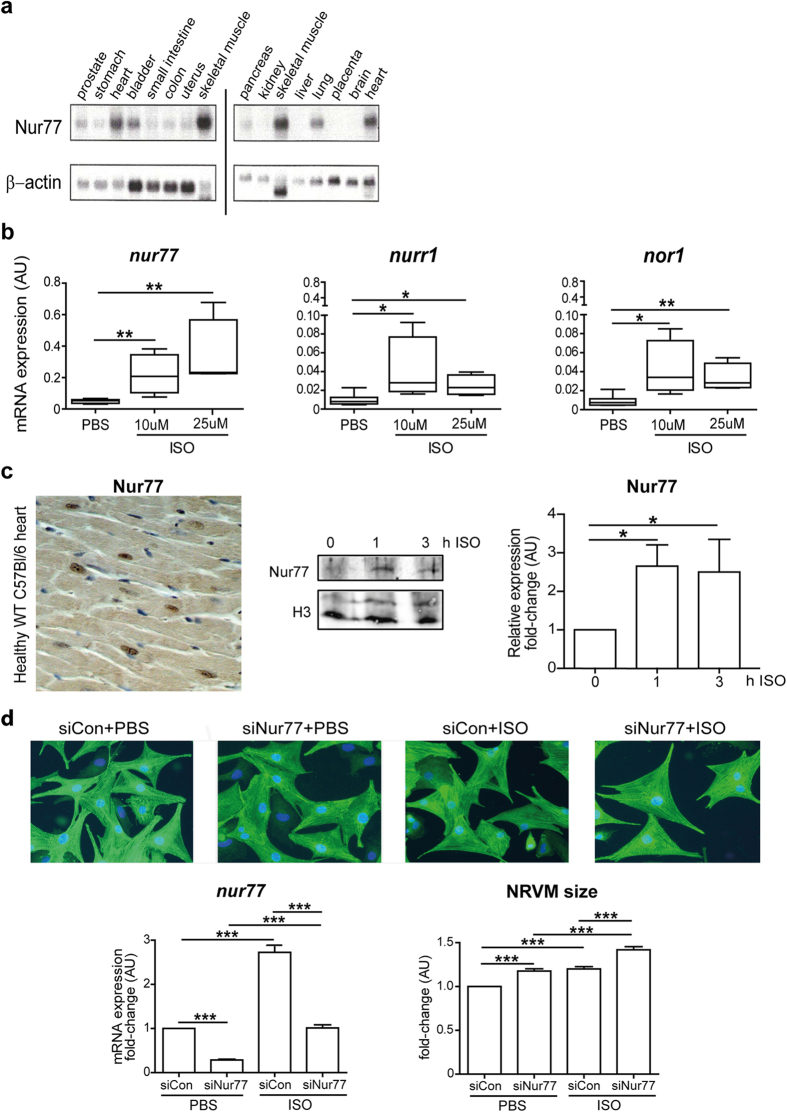
Isoproterenol increases Nur77 expression in cardiomyocytes and Nur77 knockdown enhances isoproterenol-induced cardiomyocyte hypertrophy. (**a**) Northern blot analysis of Nur77 expression in healthy human tissues. Heart and skeletal muscle exhibited highest endogenous Nur77 expression relative to loading control β-actin. Blots have been prepared under the same experimental conditions. (**b**) Significantly up-regulated NR4A gene expression in NRVMs after 1 h of isoproterenol (ISO) stimulation (n = 3), with Nur77 mRNA levels showing highest abundance. (**c**) Representative example of nuclear Nur77 protein expression in cardiomyocytes of healthy WT C57Bl/6 mice as assessed by immunohistochemistry. Photomicrograph shown at 200× magnification. Nur77 protein expression is significantly enhanced in nuclei of NRVMs after isoproterenol stimulation for 1 and 3 h (n = 4). (**d**) NRVMs are significantly larger after siRNA-mediated Nur77 knockdown (siNur77) compared to control-transfected (siCon) cells under both control conditions and 48 h of isoproterenol stimulation, as assessed by α-Actinin staining in >250 cells per group (n = 3). Photomicrographs are shown at 200× magnification. Boxplots represent median, inter-quartile range and minimum/maximum values; bars represent mean + SEM; *p < 0.05, **p<0.01, ***p < 0.001.

Nur77 is rapidly and highly expressed in many tissues upon various stimuli. However, as shown on multi-tissue Northern blots, endogenous Nur77 expression is most pronounced in healthy human heart and skeletal muscle (Fig. 1a). Given that NR4A mRNA expression in whole hearts is increased by β-adrenergic stimulation^18^,^19^, we assessed expression of Nur77, Nurr1 and NOR-1 in neonatal rat cardiomyocytes (NRVMs) upon β-adrenergic stimulation. After 1 h of stimulation with β-adrenergic agonist isoproterenol, gene expression of all three NR4A family members was robustly increased compared to PBS-stimulated cells, however Nur77 was expressed to the highest extent amongst all NR4A family members (Fig. 1b). Expression of all NR4A mRNAs was reduced back to control levels after 3 h of isoproterenol stimulation (Supplementary Fig. S1). Nuclear Nur77 protein is observed in healthy murine cardiomyocytes and its expression was markedly enhanced after 1 h and 3 h of isoproterenol stimulation (Fig. 1c). This high and rapid induction of Nur77 upon isoproterenol stimulation suggests a prominent role for Nur77 in the heart’s response to adrenergic stimulation. To see whether this has pathological implications, we assessed isoproterenol-induced cardiomyocyte hypertrophy upon Nur77 knockdown. Transfection with Nur77-specific siRNA (siNur77) resulted in markedly down-regulated Nur77 mRNA expression, and it significantly inhibited the observed isoproterenol-induced Nur77 mRNA upregulation when compared to control-transfected (siCon) cells (Fig. 1d). Interestingly, Nur77 knockdown in unstimulated NRVMs already resulted in significantly larger cardiomyocytes than siCon-transfected cells (Fig. 1d). Upon isoproterenol stimulation, siNur77 and siCon-transfected NRVMs significantly increased in size. Again, the siNur77-transfected NRVMs were significantly larger than siCon NRVMs, showing that reduced Nur77 induces a hypertrophic response in isolated cardiomyocytes.

### Nur77-KO cardiomyocytes exhibit elevated [Ca^2+^]_i_ and prolonged action potentials

**Figure 2:**
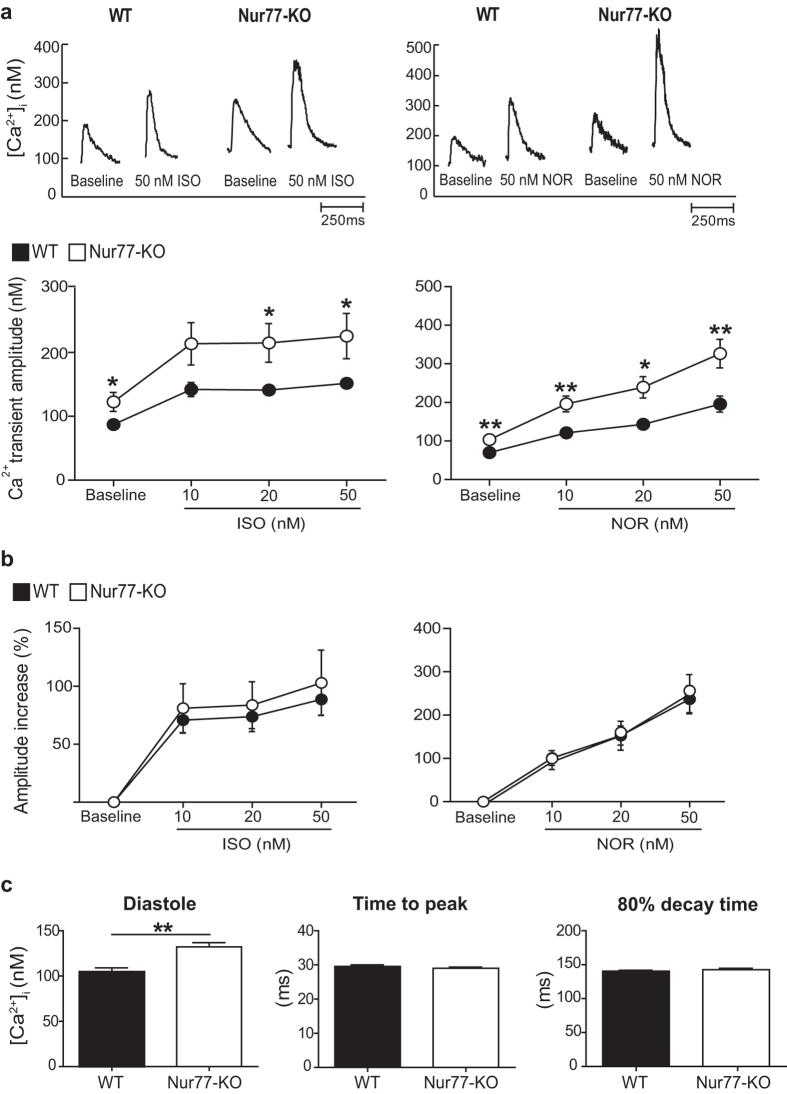
Nur77-KO cardiomyocytes exhibit altered [Ca^2+^]_i_ homeostasis. Adult cardiomyocytes were isolated from at least 3 different WT and Nur77-KO mice. Baseline measurements were performed in WT (n = 31) and Nur77-KO (n = 33) cardiomyocytes, of which 14–19 per group were subsequently stimulated with isoproterenol (ISO) or norepinephrine (NOR). (**a**) Representative [Ca^2+^]_i_ transient and amplitude quantification. [Ca^2+^]_i_ transient amplitudes were significantly higher in Nur77-KO cardiomyocytes at baseline and upon stimulation with either isoproterenol or norepinephrine. (**b**) Amplitude increase relative to baseline upon adrenergic stimulation did not differ between WT and Nur77-KO cardiomyocytes. (**c**) Basal diastolic [Ca^2+^]_i_ concentration was significantly elevated in Nur77-KO cardiomyocytes, while transient peak and decay times were unchanged compared to WT cardiomyocytes. Data presented as mean±SEM; *p < 0.05, **p < 0.01.

As Ca^2+^ is the key effector of adrenergic signalling, we analysed [Ca^2+^]_i_ transients in adult wild type (WT) and Nur77-KO cardiomyocytes in the absence or presence of the synthetic adrenergic agonist isoproterenol or the native adrenergic agonist norepinephrine. Already at baseline, Nur77-KO cardiomyocytes exhibited significantly larger [Ca^2+^]_i_ transient amplitudes compared to WT cells, a difference which was preserved upon stimulation with increasing concentrations of either isoproterenol or norepinephrine (Fig. 2a). The relative amplitude increase upon adrenergic stimulation did not significantly differ between WT and Nur77-KO cardiomyocytes (Fig. 2b). Interestingly, basal diastolic [Ca^2+^]_i_ concentrations were significantly elevated in Nur77-KO cardiomyocytes compared to WT cells, yet without differences in the time to peak and decay time of the [Ca^2+^]_i_ transient (Fig. 2c). Thus, Nur77-KO cardiomyocytes seem equally responsive to acute adrenergic stimulation when compared to WT, however exhibit significantly larger [Ca^2+^]_i_ transients and elevated diastolic [Ca^2+^]_i_ levels under basal conditions.

**Figure 3:**
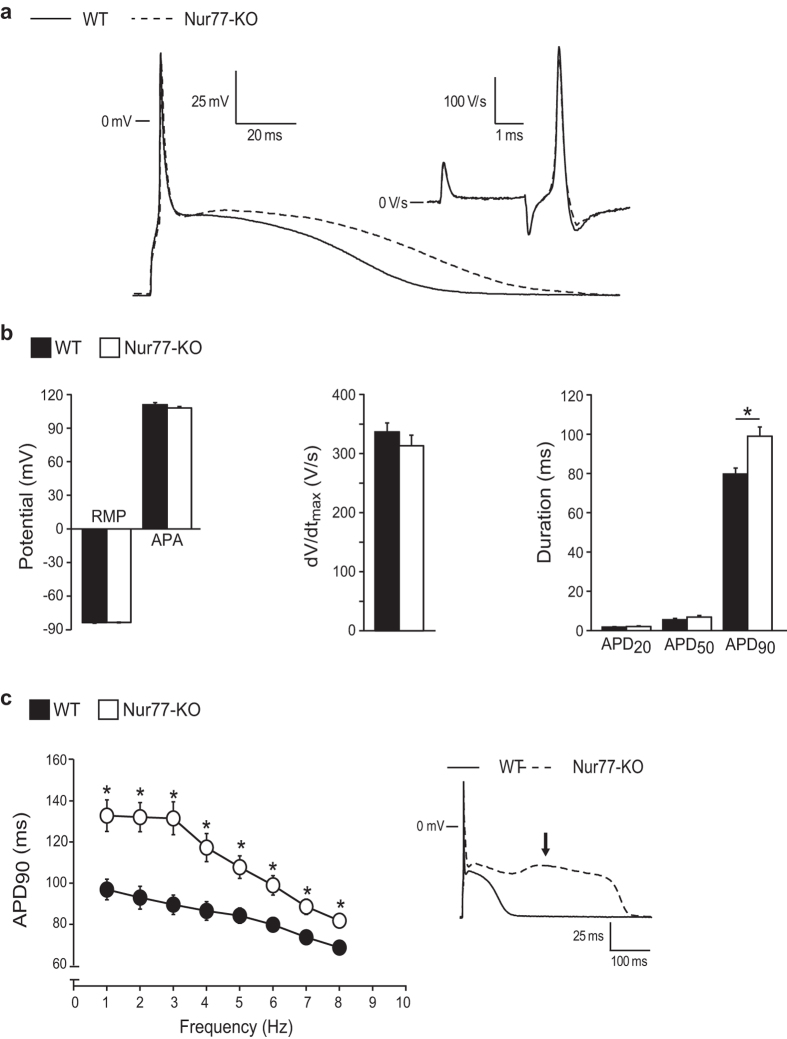
Nur77-KO cardiomyocytes exhibit prolonged action potentials. Action potential (AP) measurements were performed in WT (n = 20) and Nur77-KO (n = 18) cardiomyocytes isolated from 3 mice of each group. (**a**) Representative APs at 6 Hz. Inset shows the maximal AP upstroke velocity (dV/dt_max_). (**b**) Average AP characteristics at 6 Hz. No differences were observed in resting membrane potential (RMP), AP amplitude (APA) or AP duration (APD) at 20% and 50% repolarization. APD_90_ was significantly longer in Nur77-KO cardiomyocytes. (**c**) APD_90_ was significantly enhanced in Nur77-KO cardiomyocytes at all measured stimulation frequencies. Early after-depolarisations (inset; arrow) were observed only in a subset of Nur77-KO cardiomyocytes at 1 and 2 Hz. Data presented as mean ± SEM; *p < 0.05.

To further characterize Nur77-KO cardiomyocytes, we measured if the changes in [Ca^2+^]_i_ influence the electrophysiological properties of these cells. Figure 3a,b, show typical action potentials (APs) and average AP properties, respectively, measured at 6 Hz. No differences in resting membrane potential (RMP) and AP amplitude (APA) were observed between WT and Nur77-KO cardiomyocytes. Also, maximal AP upstroke velocity (dV/dt_max_), a measure of sodium channel availability, did not differ between the groups. This is in line with unaltered *ex vivo* conduction parameters observed between isolated, Langendorff-perfused, WT and Nur77-KO hearts (Supplementary Fig. S2). While no significant differences in AP duration at 20% and 50% of repolarization (APD_20_ and APD_50_, respectively) were observed, Nur77-KO APs display a significantly longer duration at 90% repolarization (APD_90_) compared to WT. Specifically a 24% lengthening of the APD_90_ was observed. Prolonged APs are in line with the trend towards prolonged *ex vivo* effective refractory period (ERP; p = 0.06) of Nur77-KO hearts. Significant changes in APD_90_ were evident at all measured stimulation frequencies (Fig. 3c). Interestingly, at a stimulus frequency of 1 and 2 Hz, early after-depolarisations (EADs; Fig. 3c inset; arrow) were observed in 22% of Nur77-KO cardiomyocytes, but never in WT cells (P ≤ 0.05, Fisher exact test). Taken together these data suggest a role for Nur77 in electrochemical Ca^2+^ homeostasis maintenance in cardiomyocytes.

### Expression of cardiac Ca^2+^-handling-related genes

**Figure 4:**
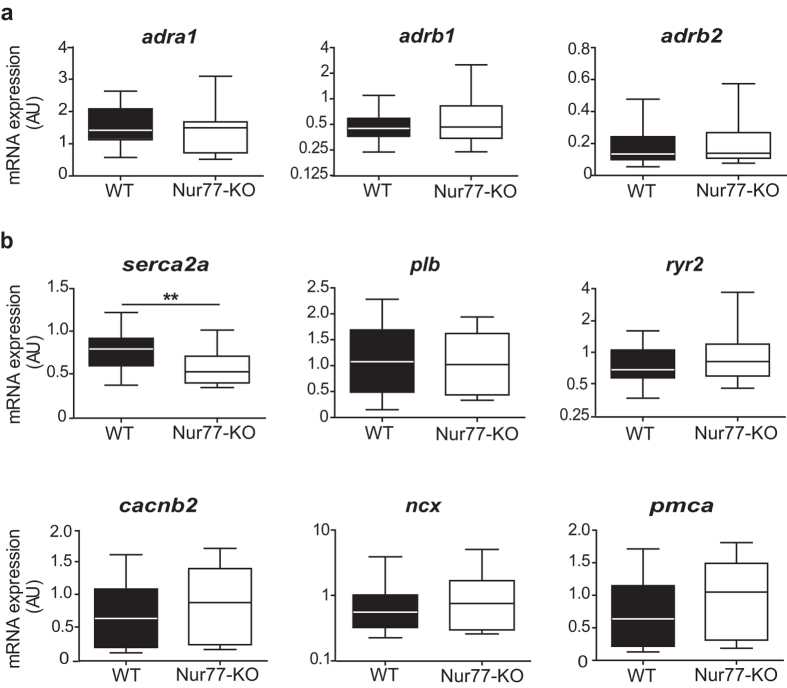
Gene expression patterns of adrenergic receptors and Ca^2+^ -handling proteins in WT and Nur77-KO hearts. RT-PCR analysis of ventricular lysates from WT and Nur77-KO mice (n = 6 each). (**a**) Expression of α- and β-adrenergic receptors did not differ between WT and Nur77-KO. *adra1*: α1-adrenergic receptor; *adrb1*: β1-adrenergic receptor; *adrb2*: β2-adrenergic receptor (**b**) *serca2a* was significantly down-regulated in Nur77-KO mice, while all other assessed Ca^2+^ -handling proteins were not differentially expressed. *serca2a*: sarco/endoplasmic Ca^2+^ ATPase; *plb*: phospholamban; *ryr2*: ryanodine receptor 2; *cacnb2*: l-type Ca^2+^ channel; *ncx*: Na^+^/Ca^2+^ exchanger; *pmca*: plasma membrane Ca^2+^ ATPase. Boxplots represent median, inter-quartile range and minimum/maximum values; **p < 0.01.

As Nur77 is a transcriptional regulator, we wondered if the altered Ca^2+^ homeostasis in Nur77-KO cardiomyocytes may be explained by differences on gene expression level. Thus, we analysed gene expression of adrenergic receptors and Ca^2+^-handling proteins in left ventricular lysates of healthy WT and Nur77-KO mouse hearts. Neither α- nor the major β-adrenergic receptor subtypes (*adra1, arb1, adrb2*) were expressed differentially in Nur77-KO left ventricles compared to WT (Fig. 4a), suggesting there is no obvious difference in endogenous cardiac adrenergic stimulation between the groups. Interestingly, only a significant down-regulation of *serca2a* gene expression was detected in Nur77-KO mice, while genes encoding for phospholamban (*plb*), the ryanodine receptor (*ryr2*), the L-type Ca^2+^ channel (*cacnb2*), the Na^+^/Ca^2+^ exchanger (*ncx*) and plasma membrane Ca^2+^ ATP-ase (*pmca*) did not differ significantly (Fig. 4b).

### Nur77 deficiency aggravates isoproterenol-induced cardiac remodelling

**Figure 5:**
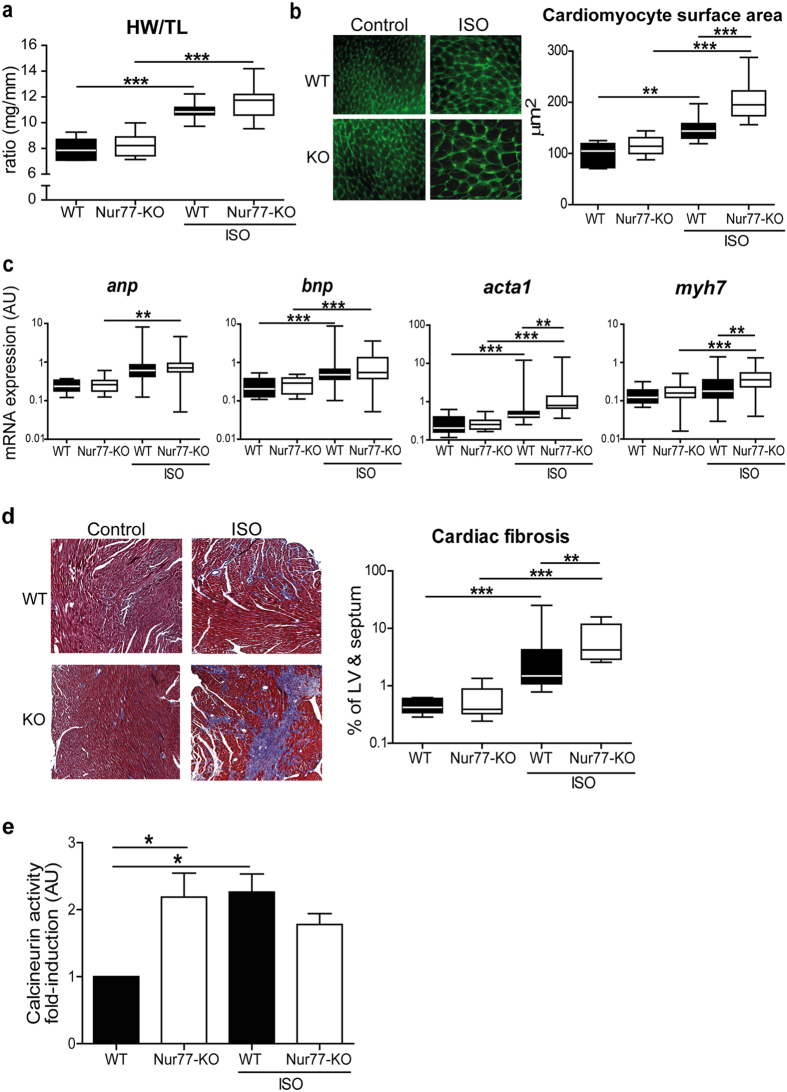
Nur77-KO mice exhibit enhanced adverse cardiac remodelling after chronic β-adrenergic stimulation. WT (n = 16) and 13 surviving Nur77-KO mice were analysed after 1 week of chronic isoproterenol (ISO) infusion. (**a**) Heart weight/tibia length (HW/TL) ratio was higher after isoproterenol stimulation and further increased in Nur77-KO mice compared to WT mice. Tibia length did not differ between WT and Nur77-KO mice. (**b**) Cardiomyocyte cross-sectional area was significantly larger in isoproterenol-treated Nur77-KO mice compared to treated WT mice, as assessed by fluorescent wheat germ agglutinin staining in >75 cells per heart. Photomicrographs are shown at 630× magnification. (**c**) RT-PCR analysis showed significant up-regulation of foetal gene expression after isoproterenol treatment. Nur77-KO mice exhibited a significant increase in *acta1* and *myh7* after isoproterenol, when compared to WT. *anp*: atrial natriuretic peptide; *bnp:* brain natriuretic peptide; *acta1*: α1 skeletal actin; *myh7*: β-myosin heavy chain. (**d**) Collagen deposition was assessed by Masson’s Trichrome staining assessed by quantitative morphometry, photomicrographs are shown at 50× magnification. The left ventricle and septum of Nur77-KO mice was affected stronger by interstitial fibrosis (blue) after isoproterenol than WT mice. (**e**) Calcineurin phosphatase activity is significantly higher in myocardium of control Nur77-KO mice. After chronic isoproterenol stimulation, calcineurin activity is significantly enhanced in WT hearts, while it does not further increase in Nur77-KO mice compared to Nur77-KO control mice (n = 3–4 in each group). Boxplots represent median, inter-quartile range and minimum/maximum values; bars represent mean+SEM; *p < 0.05, **p <0.01, ***p < 0.001.

Alteration of cardiomyocyte [Ca^2+^]_i_ has long been implied as an important regulator of maladaptive cardiac remodelling and heart failure^6^, therefore we assessed the effect of Nur77 deficiency on the development of adverse cardiac remodelling in response to chronic β-adrenergic stimulation. We implanted osmotic minipumps, delivering a relatively high dose of isoproterenol for one week, in WT and Nur77-KO mice. During the experiment, 3 out of 16 isoproterenol-treated Nur77-KO mice died, two of which had no apparent cardiac tissue damage and one mouse exhibiting cardiac rupture (tamponade). All isoproterenol-treated WT mice (n = 16) and control Nur77-KO and WT mice (n = 9 per group) survived the experiment. Although no significant changes in cardiac function were apparent after 1 week (Supplementary Fig. S3), the effectiveness of this short-term hypertrophy model is demonstrated by a higher heart weight to tibia length (HW/TL) ratio in the isoproterenol-treated mice compared to their controls (Fig. 5a). Furthermore, the surviving Nur77-KO mice exhibited a trend (p = 0.06) towards a higher HW/TL ratio when compared to the WT mice. Validation of the cardiomyocyte surface area showed that cardiomyocytes were significantly larger in isoproterenol-treated mice compared to the controls. In line with Nur77 knockdown in cultured rat cardiomyocytes, isoproterenol-treated Nur77-KO cardiomyocytes were significantly larger than in isoproterenol-treated WT mice (Fig. 5b).

Assessment of the hypertrophic foetal gene program^20^, revealed that all measured genes were up-regulated in ventricular lysates of isoproterenol-treated mice compared to the untreated controls (Fig. 5c). In addition, skeletal alpha actin (*acta-1*) and β-myosin heavy chain (*myh7*) were expressed significantly higher in isoproterenol-treated Nur77-KO mice, while there was no difference in atrial and brain natriuretic factor (*anp* and *bnp*) expression. It should be noted that both Acta-1 and Myh7 are involved in contractility, suggesting involvement of Nur77 in cardiomyocyte contractility.

Fibrosis is an important pathological feature associated with adverse cardiac remodelling^2^. In the isoproterenol-treated mice, already in the short time frame of one week, isoproterenol stimulation caused interstitial fibrosis in hearts of both WT and Nur77-KO mice, when compared to the control hearts (Fig. 5d). Upon quantification, a 3-fold induction in fibrosis was measured in the Nur77-KO mice (median 4.1%), as compared to WT mice (median 1.4%). As Nur77 has been shown to regulate apoptosis in a variety of cells, including cardiomyocytes^15^, we assessed apoptotic cells in the heart after isoproterenol-induced remodelling. However, apoptotic cardiac cells were only observed in a fraction of the mice within each group, showing no difference between WT and Nur77-KO (Supplementary Fig. S3).

Protein phosphatase calcineurin is activated by sustained [Ca^2+^]_i_ elevation^21^ and calcineurin activation has long been implied as a sufficient inducer of hypertrophic signalling in heart^22^. We hypothesized that the elevated [Ca^2+^]_i_ in Nur77-KO cardiomyocytes may lead to higher activation of calcineurin and therefore may explain the enhanced remodelling observed in Nur77-KO mice. Therefore, we assessed calcineurin activity in left ventricular lysates. Indeed, calcineurin activity is already 2-fold higher in control Nur77-KO mice compared to WT (Fig. 5e). While calcineurin activity is significantly enhanced in WT hearts after isoproterenol stimulation as expected, calcineurin was interestingly not further increased in Nur77-KO mice.

Taken together, these results demonstrate a cardioprotective role for Nur77 upon chronic β-adrenergic stimulation. However, how do these data relate to the existing Nur77 data on cardiac remodelling?

### Nur77 deficiency attenuates TAC-induced cardiac remodelling

**Figure 6:**
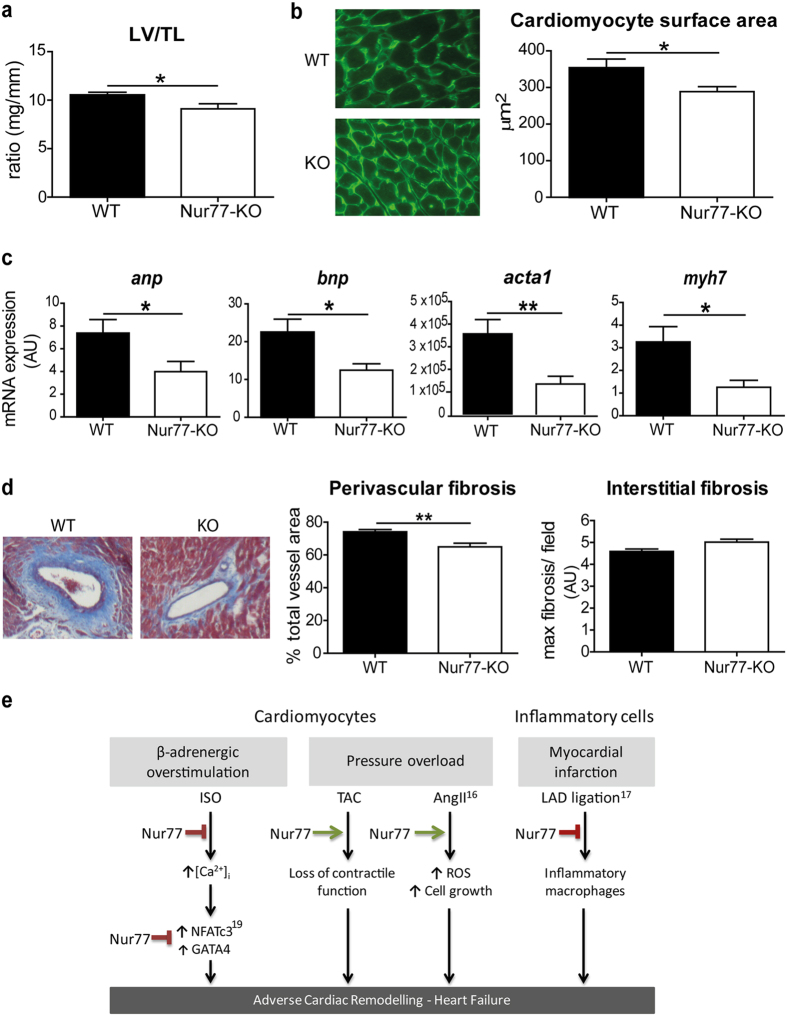
Attenuated pressure overload-induced adverse cardiac remodelling in Nur77-KO mice. WT (n = 12) and Nur77-KO (n = 11) mice were analysed after 28 days of TAC. (**a**) Left ventricle/tibia length (LV/TL) ratio was significantly lower in Nur77-KO mice after TAC than in WT mice. Tibia length did not differ between WT and Nur77-KO mice. (**b**) Cardiomyocytes from Nur77-KO mice were significantly smaller compared to cardiomyocytes from WT mice, as assessed by fluorescent wheat germ agglutinin staining in >75 cells per heart. Photomicrographs are shown at 630× magnification. (**c**) Foetal gene expression after TAC was significantly down-regulated in Nur77-KO mice, as assessed by RT-PCR. *anp*: atrial natriuretic peptide; *bnp:* brain natriuretic peptide; *acta1*: α1 skeletal actin; *myh7*: β-myosin heavy chain. (**d**) Collagen deposition as assessed by Masson’s Trichrome staining, photomicrograph shown at 100× magnification. Significantly less perivascular fibrosis was present in Nur77-KO mice compared to WT, while areas of interstitial fibrosis were not different. *p < 0.05, **p < 0.01. (**e**) Proposed mechanism for the role of Nur77 in cardiac remodelling induced by different insults. ISO: isoproterenol; TAC: transverse aortic constriction; AngII: angiotensin II; LAD: left anterior descending coronary artery; ROS: reactive oxygen species.

Very recently, it was shown that local adenoviral overexpression of Nur77 in murine hearts could reduce hypertrophy after β-adrenergic stimulation with isoproterenol^19^, supporting our findings. In two different models of pressure overload, by chronic angiotensin-II infusion^16^ or TAC^15^, up-regulation of cardiac Nur77 expression was observed. Interestingly, cardiac remodelling by angiotensin-II-induced pressure overload was attenuated in Nur77-KO mice^16^. As this is in contrast with the observations after β-adrenergic overstimulation, we subjected WT and Nur77-KO mice to TAC-induced cardiac pressure overload, to establish whether the effect of Nur77 deficiency is dependent on the type of cardiac stressor. In line with the angiotensin-II model, cardiac pressure overload in Nur77-KO mice led to attenuated adverse cardiac remodelling compared to WT after 28 days of TAC. Survival was equal between the two groups; 1 out of 13 WT and 1 out of 12 Nur77-KO mice died during the experiment. Nur77-KO mice showed a trend towards enhanced cardiac function (p = 0.10 ejection fraction, p = 0.08 fractional shortening) assessed by echocardiography (Supplementary Fig. S4). In addition to cardiac function, left ventricular weight/TL ratio was lower in Nur77-KO mice compared to WT (Fig. 6a), suggesting reduced hypertrophy. Indeed, the mean cardiomyocyte surface area was significantly smaller in Nur77-KO mice (Fig. 6b). Moreover, expression of *anp, bnp, acta-1* and *myh7* were all significantly lower in Nur77-KO ventricular tissue (Fig. 6c). As TAC induces cardiac pressure overload, we assessed perivascular and interstitial fibrosis separately. Interestingly, perivascular fibrosis was significantly higher in WT mice compared to Nur77-KO mice, whereas no significant difference was observed in interstitial collagen deposition (Fig. 6d). As in the isoproterenol model, no difference in the number of apoptotic cells in WT and Nur77-KO hearts was found after TAC (Supplementary Fig. S4).

In conclusion, our data demonstrate that Nur77 plays a detrimental role in the heart upon TAC-induced pressure overload, in contrast to its role in isoproterenol-induced cardiac remodelling. Taking together our data on Nur77 in the heart with existing data, we propose the following scheme on the function of Nur77 in different cardiac insult models, leading to heart failure (Fig. 6e).

## Discussion

Essential roles for Nur77 have been demonstrated regarding atherosclerosis^10^,^11^,^23^, cancer^24^ and metabolic disease^25^. However, knowledge on the functional role of Nur77 in the cardiac setting is limited. The abundant endogenous expression of the NR4A family of nuclear receptors, including Nur77, in healthy murine heart tissue was previously described by Myers *et al.*^18^. Here, we demonstrate that also in the healthy human heart, endogenous Nur77 expression is relatively high as compared to other tissues.

A number of studies describe enhanced expression of Nur77 in the diseased heart^15^,^16^,^17^,^19^. Transient up-regulation of cardiac Nur77 mRNA and protein has been shown in animal models of pressure-overload induced by TAC^15^, chronic angiotensin-II infusion^16^, as well as in ischemia following myocardial infarction^15^. Nur77-KO mice and rats with cardiac-specific Nur77 knock-down exhibited attenuated cardiac remodelling after chronic angiotensin-II infusion. Angiotensin-II induces cardiac hypertrophy by increasing systemic blood pressure in conjunction with increased signalling of the angiotensin-II type I receptor on cardiomyocytes^26^. Nur77 is considered detrimental in this model by activating mTORC1, leading to cardiac hypertrophy by enhancement of protein synthesis, cardiomyocyte growth and reactive oxygen species (ROS) production^16^. In cultured cardiomyocytes, a ROS challenge was shown to cause Nur77 translocation to mitochondria, causing cytochrome c release and subsequent apoptosis^15^. In the same study, mitochondrial translocation of Nur77 was observed after ischemia/reperfusion injury in the heart. In our murine studies, apoptosis did not seem to play a key role in the observed pathologies. In contrast, Hilgendorf *et al.*^17^ observed after myocardial infarction, that Nur77-KO mice exhibit enhanced adverse cardiac remodelling with increased inflammation, fibrotic scar size and ventricular dysfunction^17^.

Rapid up-regulation of Nur77 in whole mouse hearts was previously reported after a single intraperitoneal injection of isoproterenol^18^,^19^. Here, we show that both Nur77 mRNA and nuclear protein are rapidly and highly up-regulated in isolated cardiomyocytes in response to isoproterenol stimulation. To assess Nur77 function, knockdown of Nur77 in cultured cardiomyocytes revealed that Nur77 deficiency caused a hypertrophic response, which was further enhanced upon isoproterenol stimulation. Very recently, Yan *et al.*^19^ demonstrated, in accordance with our data, attenuated cardiomyocyte hypertrophy after isoproterenol stimulation when overexpressing Nur77. In addition, adverse cardiac remodelling in mice was reduced by cardiac-specific Nur77 overexpression. They reveal an interaction of Nur77 with the pro-hypertrophic transcription factors NFATc3 and GATA-4, of which NFATc3 is regulated by calcineurin. In our study, we show that the adverse cardiac remodelling we observe in Nur77-KO mice after chronic isoproterenol treatment, may be initiated by elevated cardiomyocyte [Ca^2+^]_i_ and downstream calcineurin, since diastolic [Ca^2+^]_I_ and [Ca^2+^]_i_ transient amplitudes are already significantly elevated in unstimulated Nur77-KO cardiomyocytes and calcineurin activity is enhanced in healthy Nur77-KO myocardium.

Elevation of diastolic [Ca^2+^]_i_ is a well-known feature of overtly failing cardiomyocytes, accompanied by decreased [Ca^2+^]_i_ transient amplitudes and prolonged decay times^27^. This diastolic Ca^2+^ accumulation is usually associated with diminished sarcoplasmic sequestration, often by lowered Serca function^28^. Indeed we observed significantly lower Serca2a mRNA expression in Nur77-KO hearts. However, as transient decay times were not prolonged and amplitudes were not diminished, Ca^2+^ sequestration does not seem to be impaired in Nur77-KO cardiomyocytes. To shed light on the mechanism behind the elevated diastolic and systolic [Ca^2+^]_i_ in Nur77-KO cardiomyocytes, the different mechanisms which facilitate cytosolic Ca^2+^ influx and efflux must be assessed. Higher sarcoplasmic Ca^2+^ load, increased sarcoplasmic Ca^2+^ leak due to enhanced open probability of RyR channels, increased L-type Ca^2+^ current, decreased expression or enhanced reverse mode activity of NCX and cytosolic Na^+^ accumulation have all been shown to interplay with elevated diastolic and systolic Ca^2+^ (as reviewed previously^29^). Electrophysiological characterization revealed that cardiac conduction was unaffected in Nur77-KO hearts, but Nur77-KO cardiomyocytes displayed prolonged APs and EADs. Both prolonged APs and EADs may form pro-arrhythmic substrates^30^, possibly explaining the two dead Nur77-KO mice in our isoproterenol experiment, which did not reveal an obvious cause of death. Prolonged APs and EADs may occur upon reduction of repolarization reserve by either increased L-type Ca^2+^ current or reduced potassium (K^+^) current, or a combination of both^31^,^32^. Nur77 may regulate the K^+^ channel, I_K,slow2_, encoded by the Kv2.1 gene (*kcnb1),* since a mutation in a potential Nur77 DNA-binding site in the *kcnb1* promoter reduced its activity^33^. Decreased *kcnb1* levels have been reported in cardiac disease^34^,^35^. On the other hand, an increase in L-type Ca^2+^ current in Nur77-KO cardiomyocytes would be in line with the larger [Ca^2+^]_i_ transient amplitudes. In neuronal cells, K^+^Cl^−^-induced membrane depolarization enhances [Ca^2+^]_i_ rises with subsequent activation of calcineurin^36^,^37^. Downstream of calcineurin, cAMP response element binding protein (CREB) subsequently stimulates expression of Nur77^38^. Expression of NR4A family member Nurr1 is also regulated by calcineurin and this induction was inhibited upon L-type Ca^2+^ channel blockade^39^. Taken together, we hypothesize that Nur77 may exert a feedback mechanism on [Ca^2+^]_i_ elevations and calcineurin activity.

In line with our isoproterenol experiment, Nur77-KO mice exhibited a worsened outcome after myocardial infarction^17^. However, this effect was largely attributed to deficiency of reparative Ly6C-low monocytes in the Nur77-KO mice^17^, while potential changes in cardiomyocytes were not taken into account. Given that Nur77 is essential for the development of Ly6C-low monocytes^40^ and that these cells are instrumental to repair cardiac damage and resolve fibrosis^41^, the enhanced cardiac damage observed in the Nur77-KO mice in our isoproterenol model may partially be explained by Ly6C-low monocyte deficiency. Interestingly, the absence of Ly6C-low monocytes does not seem to affect cardiac damage after TAC in a negative manner, supporting the notion that Nur77 is also important in cardiac cells.

The Nur77-KO cardiomyocyte phenotype appears unfavourable, yet, challenging Nur77-KO mice with different cardiac stressors resulted in opposite remodelling outcomes. In line with published data^16^, we also observed that Nur77 has an adverse function in cardiac remodelling caused by pressure overload, while we reveal a protective role for Nur77 in cardiac remodelling caused by β-adrenergic overstimulation. While activation of β-adrenergic signalling may be present as a compensatory mechanism upon pressure overload, the role of Nur77 seems to be dependent on the dominant cardiac stressor. We hypothesize that increased [Ca^2+^]_i_ availability in Nur77-KO cardiomyocytes preserves contractility, which may compensate for the increased pressure, leading to improved outcome after TAC. Chronic isoproterenol stimulation modulates Ca^2+^ handling by increasing cardiomyocyte AP duration, enhancing reverse Na^+^/Ca^2+^-exchange and decreasing K^+^ current density (reviewed in^42^). In Nur77-KO cardiomyocytes, where basal [Ca^2+^]_i_ is already relatively high, we hypothesize that chronic β-adrenergic stimulation induces [Ca^2+^]_i_ abnormalities more easily.

Since only limited changes were observed in cardiac function in both models as assessed by echocardiography, our study contributes to the understanding of early stages of cardiac remodeling, when intervention to prevent the heart from eventual failure is still feasible.

In conclusion, we have shown for the first time that Nur77 deficiency leads to elevated intracellular Ca^2+^ levels and prolonged action potentials in cardiomyocytes and enhanced calcineurin activity in Nur77-KO hearts, however resulting in opposite cardiac remodelling outcome after different types of cardiac insults. Modulation of Nur77 may be a novel therapeutic target in managing adverse myocardial remodelling and heart failure, discriminating between cardiac stressors. Our study supports the assumption that knowing the induction mechanism of cardiac remodelling in patients is crucially important before deciding on a therapeutic strategy.

## Methods

### Northern blot analysis

Human multiple tissue poly(A)^+^ RNA blots were purchased from CLONTECH (Palo Alto, CA, USA) and hybridized according to manufacturer’s protocol. Detection was performed using a fragment of human Nur77 cDNA (base pairs 864 to 1905) and the supplied human β-actin cDNA probe for normalisation.

### Cell isolation and culture

Neonatal rat ventricular myocytes (NRVMs) were isolated as previously described^43^. In short, hearts from 1–3 day old Wistar rats were excised, atria were removed, and hearts were cut into 4–6 pieces. The pieces were left overnight to rotate at 4 °C in HBSS (Invitrogen, Carlsbad, CA, USA) containing 1 mg/ml trypsin (Affymetrix, Santa Clara, CA, USA). The next day, cells were dissociated using 1 mg/ml collagenase (Worthington, Lakewood, NJ, USA) in HBSS. Cells were collected and resuspended in M199 culture medium, supplemented with 1% HEPES, 1% NEAA, 2 mg/L vitamin B12, 3.5 g/L glucose, and antibiotics (all from Invitrogen), and pre-plated to separate myocytes from fibroblasts. After 2 hours, non-adherent cells were collected, counted and plated on fibronectin-coated plates. NRVMs were serum-starved for 24 h in Dulbecco’s modified Eagle medium (DMEM) supplemented with penicillin/streptavidin (Invitrogen) before experiments.

### Protein isolation and Western blotting

NRVMs were stimulated with 25 µm isoproterenol (Sigma-Aldrich, St. Louis, MO, USA) or equivalent volume of PBS for indicated time points. Nuclear protein was isolated from NRVMs using the NE-PER Nuclear and Cytoplasmic extraction kit (Thermo Scientific, Rockford, IL, USA) according to manufacturer’s protocol. Lysates were boiled, separated by SDS-PAGE and transferred onto PVDF membranes. After transfer, membranes were blocked with Odyssey blocking buffer (LI-COR, Lincoln, NB, USA) and incubated with anti-Nur77 (Santa Cruz, Dallas, TX, USA) and anti-H3 (Abcam, Cambridge, UK) for normalisation. Anti-rabbit IRDye800 was used as secondary antibody (LI-COR). Blots were scanned and quantified using the LI-COR Odyssey Infrared Imaging System.

### Nur77 knockdown and NRVM size assessment

NRVMs were transfected with a final concentration of 175 nM Nur77 siRNA (SMARTpool, Dharmacon, Lafayette, CO, USA) or a non-targeting control siRNA for 6 h using Lipofectamine RNAiMAX (Invitrogen) according to manufacturer’s instructions. For assessment of Nur77 knockdown, NRVMs were cultured for 48 h after transfection and were subsequently stimulated with 25 µm isoproterenol or equivalent volume of PBS for 1 h. For cell size experiments, NRVMs were stimulated with 25 µm isoproterenol or PBS for 48 h after transfection. Cells were fixed with 4% paraformaldehyde (Roth, Karlsruhe, Germany) and stained with anti-α-Actinin antibody (Sigma Aldrich), Hoechst and anti-mouse AlexaFluor488-conjugated secondary antibody (Molecular Probes, Eugene, OR, USA). Size was measured in ≥250 Actinin-positive cells per group using ImageJ software (U.S. National Institutes of Health, Bethesda, Maryland, USA).

### Mice

All animal housing, care and procedures were approved by the institutional Animal Experimental Committee for Animal Welfare in accordance with the Directive 2010/63/EU of the European Parliament. Mice used for cardiomyocyte isolations and experimental procedures are C57Bl/6 WT or Nur77-KO mice in C57Bl/6 background, all 10–12 week-old males.

### [Ca^2+^]_i_ transient measurements

Left ventricular cardiomyocytes from WT and Nur77-KO mice were isolated by enzymatic dissociation^44^ and loaded with fluorescent Ca^2+^ probe Indo-1-AM (Molecular Probes, Eugene, OR, USA) as described previously^45^. Using 4 Hz stimulation frequency, baseline [Ca^2+^]_i_ was measured for 1 min, followed by measurements directly upon stimulation with increasing concentrations of isoproterenol (Sigma) or norepinephrine (Centrafarm, Etten-Leur, The Netherlands).

### Action potential measurements

Action potentials were recorded in unstimulated, single cardiomyocytes isolated from WT and Nur77-KO mice, by the amphotericin-perforated patch-clamp technique using an Axopatch 200B amplifier (Molecular Devices, USA) as described previously^46^.

### *Ex vivo* assessment of cardiac conduction parameters

Atrio-ventricular and ventricular conduction parameters were investigated in isolated, Langendorff-perfused WT and Nur77-KO mice hearts using optical mapping, as described previously^46^.

### Chronic isoproterenol stimulation *in vivo*

Osmotic minipumps (Alzet, Cupertino, CA, USA) were implanted subcutaneously in WT or Nur77-KO mice. The procedure was performed under isoflurane anaesthesia (4% isoflurane for induction, 2.5% isoflurane and O_2_ for maintenance of anaesthesia; Baxter, Illinois, United States) with subcutaneously-administered Carprofen (4 mg/kg) as a local analgetic. The osmotic minipumps continuously released isoproterenol (Sigma) dissolved in saline, in a dose of 60 mg/kg/day. Control mice received minipumps containing saline only. After 7 days the mice were euthanized through a lethal dose of intraperitoneal-injected ketamine (238 mg/kg)/xylazine (102 mg/kg). Prior to and 6 days after implantation of the osmotic minipumps, transthoracic echocardiography was performed in isoflurane-anaesthetized mice (4% isoflurane for induction, 2.5% isoflurane and O_2_ for maintenance of anaesthesia). Using the Vevo770 imaging system equipped with a 30-MHz linear array transducer (VisualSonics Inc, Toronto, Canada), a two-dimensional short-axis view of the left ventricle was obtained at the level of the papillary muscles and 2D M-mode tracings were recorded. Left ventricular ejection fraction and fractional shortening were calculated using the following formulas: LVEF% = (LVvol;d-LVvol;s)/(LVvol;d)*100; LVFS% = (LVID;d-LVID;s)/(LVID;d)*100.

### Transverse aortic constriction (TAC)

TAC surgeries and subsequent cardiac function assessment was performed as described previously^47^. In brief, TAC was performed in WT or Nur77-KO mice. The procedure was performed under isoflurane anaesthesia (4% isoflurane for induction, 2.5% isoflurane and O_2_ for maintenance of anaesthesia) with subcutaneously-injected Buprenorphine (0.05 mg/kg) as local and post-operative analgetic. After 28 days, 2D M-mode echocardiography (Aloka SSD 4000 echo device, 12 MHz transducer; Aloka, Tokyo, Japan) was performed under isoflurane anaesthesia, where after the mice were euthanized by 100% CO_2_ ventilation.

### Histological analysis

Ventricles were fixed in 4% formaldehyde for 24 h, embedded in paraffin, sectioned (7 μm) and mounted on Starfrost glass slides (Thermo Scientific). Nur77 protein expression was assessed by immunohistochemistry using anti-Nur77 antibody (Santa Cruz) and anti-rabbit-IgG-HRP polymer (BrightVision, ImmunoLogic, Duiven, The Netherlands). Antigen detection was performed by development with diaminobenzidine tetrachloride (DAB; Immunologic) and slides were imaged using Leica QWin V3 software (Leica Mircosystems, Cambridge, UK).

To assess cardiomyocyte cross-sectional area, cardiac tissue was stained with AlexaFluor488-conjugated wheat germ agglutinin (Molecular Probes, Eugene, OR, USA) and DAPI. Cardiomyocytes were included in size measurement if they were transversely cross-sectioned and revealed thin, intact cell borders and visible nuclei located in proximity of the cell centre. Per mouse, ≥75 cardiomyocytes were measured using ImageJ software to acquire a proper representation of the tissue. Collagen quantification was performed on Masson’s Trichrome-stained (Sigma-Aldrich) sections, using Leica QWin V3 software.

Apoptotic cells were assessed by TUNEL assay using the *In Situ* Cell Death Detection Kit (Roche, Indianapolis, IN, USA) according to manufacturer’s instructions and quantified as number of TUNEL+ cells by fluorescence microscopy.

### RNA isolation and real time-PCR

Total RNA was isolated from NRVMs using the Aurum Total RNA mini kit (BioRad Laboratories, Veenendaal, The Netherlands) and total RNA from cardiac tissue was isolated using the RNeasy Fibrous Tissue Mini kit (Qiagen, Frederick, MD, USA), all according to manufacturer’s instructions. cDNA was synthesized with the iScript cDNA Synthesis kit and real-time PCR was performed using iQ SYBR Green Supermix and was measured with the MyIQ system (all from BioRad). Specific primer sequences are listed in Table 1. mRNA levels were normalized for the combined value of ribosomal protein P0 (*p0*) and ribosomal protein L13 (*rpl13*) housekeeping genes.

### Calcineurin activity assay

Protein was isolated from snap-frozen murine left ventricles using lysis buffer supplied in the calcineurin activity assay kit (Enzo Life Sciences, Exeter, UK). Calcineurin phosphatase activity was measured in 5 ug protein from each lysate, according to the manufacturer’s instructions.

### Statistical analysis

All data analyses were performed using GraphPad Prism 5 (La Jolla, CA, USA). Data distribution was tested with Kolmogorov-Smirnov normality test. Accordingly, data is presented as mean ± SEM and tested with Student’s t-test, two-way repeated measures ANOVA or Fisher’s exact test. In case of a failed normality test, data is presented as boxplots with whiskers for minimum/maximum values and tested with Mann-Whitney-U test. Holm-Bonferonni or Newman-Keuls test post-hoc correction was used when appropriate.

## Additional Information

**How to cite this article**: Medzikovic, L. *et al.* Orphan nuclear receptor Nur77 affects cardiomyocyte calcium homeostasis and adverse cardiac remodelling. *Sci. Rep.* **5**, 15404; doi: 10.1038/srep15404 (2015).

## Supplementary Material

Supplementary Information

## Figures and Tables

**Table 1:** Primer sequences.

**Gene**	**Forward**	**Reverse**
*acta1 Mus musculus*	GGCCCCTCCATTGTGCACCG	GCCACCCTGCAACCACAGCA
*adra1 Mus musculus*	CGGTCATCCTGGTCATGTACT	TACAATGCCCAAGGTTTTGGC
*adrb1 Mus musculus*	CTCATCGTGGTGGGTAACGTG	ACACACAGCACATCTACCGAA
*adrb2 Mus musculus*	GAGCACAAAGCCCTCAAGAC	GTTGACGTAGCCCAACCAGT
*anp Mus musculus*	ATCACCCTGGGCTTCTTCCT	TGTTGGACACCGCACTGTAC
*bnp Mus musculus*	GAGGTCACTCCTATCCTCTGG	GCCATTTCCTCCGACTTTTCTC
*cacnb2 Mus musculus*	GTCCTTGACGCGGATACAAT	CAGCTGCTACCATCTGGACA
*myh7 Mus musculus*	ACTGTCAACACTAAGAGGGTCA	TTGGATGATTTGATCTTCCAGGG
*ncx Mus musculus*	CGAGACTGTGTCGAACCTGA	TCAGGGACCACGTAAACACA
*nr4a1 Rattus norvegicus*	TGTTGCTAGAGTCCGCCTTT	CAGTGATGAGGACCAGAGCA
*nr4a2 Rattus norvegicus*	CCCAGTGGAGGGTAAACTCA	AATGCAGGAGAAGGCAGAAA
*nr4a3 Rattus norvegicus*	TCGCTTCCACATACAAGTGC	AAGGGTTCTACAGGGCAGGT
*p0 Mus musculus*	GGACCCGAGAAGACCTCCTT	GCACATCACTCAGAATTTCAATGG
*p0 Rattus norvegicus*	CTCAGTGCCTCACTCCATCA	CTTCCTTTGCTTCGACCTTG
*plb Mus musculus*	CCTTCCTGGCATAATGGAAA	ATGCAGATCAGCAGCAGACA
*pmca1 Mus musculus*	GATCCTCTTGTCGGTGGTGT	CCGTACTTCACTTGGGCAAT
*rpl13a Mus musculus*	GGGCAGGTTCTGGTATTGGAT	GGCTCGGAAGTGGTAGGGG
*rpl13a Rattus norvegicus*	AAAAAGGAGAAGGCCAGAGC	CCGCGCATTATTTCTTCTTC
*ryr2 Mus musculus*	GCGAGCTGGCTACTATGACC	CGTTGCTAATGCTCACGAAA
*serca2a Mus musculus*	TGTGTAATGCCCTCAACAGC	AGCGGTGTGATCTGGAAAAT

*acta1*: α1 skeletal actin; *adra1*: α1-adrenergic receptor; *adrb1*: β1-adrenergic receptor; *adrb2*: β2-adrenergic receptor; *anp*: atrial natriuretic peptide; *bnp:* brain natriuretic peptide; *cacnb2*: l-type Ca^2+^ channel, β2; *myh7*: β-myosin heavy chain; *ncx*: Na^+^/Ca^2+^ exchanger; *nr4a1*: Nur77; *nr4a2*: Nurr1; *nr4a3*; NOR-1; *p0*: ribosomal protein p0; *plb*: phospholamban; *pmca*: plasma membrane Ca^2+^ ATPase; *rpl13a*: ribosomal protein l13; *ryr2*: ryanodine receptor 2; *serca2a*: sarco/endoplasmic Ca^2+^ ATPase.

## References

[b1] Lymperopoulos A., Rengo G. & Koch W. J. Adrenergic nervous system in heart failure: pathophysiology and therapy. Circ. Res. 113, 739–53 (2013). 23989716 10.1161/CIRCRESAHA.113.300308 PMC3843360

[b2] Hill J. A. & Olson E. N. Cardiac plasticity. N. Engl. J. Med. 358, 1370–80 (2008). 18367740 10.1056/NEJMra072139

[b3] Barrese V. & Taglialatela M. New advances in beta-blocker therapy in heart failure. Front. Physiol. 4, 323 (2013). 24294204 10.3389/fphys.2013.00323 PMC3827547

[b4] Roger V. L. Epidemiology of heart failure. Circ. Res. 113, 646–59 (2013). 23989710 10.1161/CIRCRESAHA.113.300268 PMC3806290

[b5] Bers D. M. Cardiac excitation-contraction coupling. Nature 415, 198–205 (2002). 11805843 10.1038/415198a

[b6] Zarain-Herzberg A., Fragoso-Medina J. & Estrada-Avilés R. Calcium-regulated transcriptional pathways in the normal and pathologic heart. IUBMB Life 63, 847–55 (2011). 21901815 10.1002/iub.545

[b7] Luo M. & Anderson M. E. Mechanisms of altered Ca^2+^ handling in heart failure. Circ. Res. 113, 690–708 (2013). 23989713 10.1161/CIRCRESAHA.113.301651 PMC4080816

[b8] Arkenbout E. K. *et al.* Protective function of transcription factor TR3 orphan receptor in atherogenesis: decreased lesion formation in carotid artery ligation model in TR3 transgenic mice. Circulation 106, 1530–5 (2002). 12234960 10.1161/01.cir.0000028811.03056.bf

[b9] Bonta P. I. *et al.* Nuclear receptor Nur77 inhibits vascular outward remodelling and reduces macrophage accumulation and matrix metalloproteinase levels. Cardiovasc. Res. 87, 561–8 (2010). 20189954 10.1093/cvr/cvq064

[b10] Hamers A. A. J. *et al.* Bone marrow-specific deficiency of nuclear receptor Nur77 enhances atherosclerosis. Circ. Res. 110, 428–38 (2012). 22194623 10.1161/CIRCRESAHA.111.260760

[b11] Hanna R. N. *et al.* NR4A1 (Nur77) deletion polarizes macrophages toward an inflammatory phenotype and increases atherosclerosis. Circ. Res. 110, 416–27 (2012). 22194622 10.1161/CIRCRESAHA.111.253377 PMC3309661

[b12] Shao Q. *et al.* Nuclear receptor Nur77 suppresses inflammatory response dependent on COX-2 in macrophages induced by oxLDL. J. Mol. Cell. Cardiol. 49, 304–11 (2010). 20381497 10.1016/j.yjmcc.2010.03.023

[b13] Bonta P. I. *et al.* Nuclear receptors Nur77, Nurr1, and NOR-1 expressed in atherosclerotic lesion macrophages reduce lipid loading and inflammatory responses. Arterioscler. Thromb. Vasc. Biol. 26, 2288–94 (2006). 16873729 10.1161/01.ATV.0000238346.84458.5d

[b14] Zeng H. *et al.* Orphan nuclear receptor TR3/Nur77 regulates VEGF-A-induced angiogenesis through its transcriptional activity. J. Exp. Med. 203, 719–29 (2006). 16520388 10.1084/jem.20051523 PMC2118245

[b15] Mitochondrial translocation of Nur77 mediates cardiomyocyte apoptosis. Cheng Z. *et al.* Mitochondrial translocation of Nur77 mediates cardiomyocyte apoptosis. Eur. Heart J. 32, 2179–88 (2011). 21228009 10.1093/eurheartj/ehq496 PMC3164102

[b16] Wang R.-H. *et al.* The orphan receptor TR3 participates in angiotensin II-induced cardiac hypertrophy by controlling mTOR signalling. EMBO Mol. Med. 5, 137–48 (2013). 23197407 10.1002/emmm.201201369 PMC3569659

[b17] Hilgendorf I. *et al.* Ly-6Chigh monocytes depend on Nr4a1 to balance both inflammatory and reparative phases in the infarcted myocardium. Circ. Res. 114, 1611–22 (2014). 24625784 10.1161/CIRCRESAHA.114.303204 PMC4017349

[b18] Myers S., Eriksson N., Burow R., Wang S.-C. M. & Muscat, G. E. O. Beta-adrenergic signaling regulates NR4A nuclear receptor and metabolic gene expression in multiple tissues. Mol. Cell. Endocrinol. 309, 101–8 (2009). 19465082 10.1016/j.mce.2009.05.006

[b19] Yan G. *et al.* Orphan Nuclear Receptor Nur77 Inhibits Cardiac Hypertrophic Response to Beta-Adrenergic Stimulation. Mol. Cell. Biol. (2015), doi: 10.1128/MCB.00229–15. PMC4561731 26195821

[b20] Kuwahara K., Nishikimi T. & Nakao K. Transcriptional regulation of the fetal cardiac gene program. J. Pharmacol. Sci. 119, 198–203 (2012). 22786561 10.1254/jphs.12r04cp

[b21] Dolmetsch R. E., Lewis R. S., Goodnow C. C. & Healy J. I. Differential activation of transcription factors induced by Ca^2+^ response amplitude and duration. Nature 386, 855–8 (1997). 9126747 10.1038/386855a0

[b22] Molkentin J. D. *et al.* A calcineurin-dependent transcriptional pathway for cardiac hypertrophy. Cell 93, 215–28 (1998). 9568714 10.1016/s0092-8674(00)81573-1 PMC4459646

[b23] Van Tiel C. M. & de Vries C. J. M. NR4All in the vessel wall. J. Steroid Biochem. Mol. Biol. 130, 186–93 (2012). 21277978 10.1016/j.jsbmb.2011.01.010

[b24] To S. K. Y., Zeng J.-Z. & Wong A. S. T. Nur77: a potential therapeutic target in cancer. Expert Opin. Ther. Targets 16, 573–85 (2012). 22537097 10.1517/14728222.2012.680958

[b25] Pearen M. A. & Muscat G. E. O. Minireview: Nuclear hormone receptor 4A signaling: implications for metabolic disease. Mol. Endocrinol. 24, 1891–903 (2010). 20392876 10.1210/me.2010-0015 PMC5417389

[b26] Paradis P., Dali-Youcef N., Paradis F. W., Thibault G. & Nemer M. Overexpression of angiotensin II type I receptor in cardiomyocytes induces cardiac hypertrophy and remodeling. Proc. Natl. Acad. Sci. USA 97, 931–6 (2000). 10639182 10.1073/pnas.97.2.931 PMC15433

[b27] Piacentino V. *et al.* Cellular basis of abnormal calcium transients of failing human ventricular myocytes. Circ. Res. 92, 651–8 (2003). 12600875 10.1161/01.RES.0000062469.83985.9B

[b28] Hasenfuss G. *et al.* Relation between myocardial function and expression of sarcoplasmic reticulum Ca(2+)-ATPase in failing and nonfailing human myocardium. Circ. Res. 75, 434–42 (1994). 8062417 10.1161/01.res.75.3.434

[b29] Louch W. E., Sheehan K. A. & Wolska B. M. Methods in cardiomyocyte isolation, culture, and gene transfer. J. Mol. Cell. Cardiol. 51, 288–98 (2011). 21723873 10.1016/j.yjmcc.2011.06.012 PMC3164875

[b30] el-Sherif N., Caref E. B., Yin H. & Restivo M. The electrophysiological mechanism of ventricular arrhythmias in the long QT syndrome. Tridimensional mapping of activation and recovery patterns. Circ. Res. 79, 474–92 (1996). 8781481 10.1161/01.res.79.3.474

[b31] Benitah J.-P., Alvarez J. L. & Gómez A. M. L-type Ca(2+) current in ventricular cardiomyocytes. J. Mol. Cell. Cardiol. 48, 26–36 (2010). 19660468 10.1016/j.yjmcc.2009.07.026

[b32] Rozanski G. J. Physiological remodelling of potassium channels in the heart. Cardiovasc. Res. 93, 218–9 (2012). 22168994 10.1093/cvr/cvr340

[b33] Roder K. & Koren G. The K^+^ channel gene, Kcnb1: genomic structure and characterization of its 5′-regulatory region as part of an overlapping gene group. Biol. Chem. 387, 1237–46 (2006). 16972792 10.1515/BC.2006.153

[b34] Qin D. *et al.* Downregulation of K(+) channel genes expression in type I diabetic cardiomyopathy. Biochem. Biophys. Res. Commun. 283, 549–53 (2001). 11341759 10.1006/bbrc.2001.4825

[b35] Huang B., Qin D. & El-Sherif N. Early down-regulation of K^+^ channel genes and currents in the postinfarction heart. J. Cardiovasc. Electrophysiol. 11, 1252–61 (2000). 11083246 10.1046/j.1540-8167.2000.01252.x

[b36] Yoon J. K. & Lau L. F. Transcriptional activation of the inducible nuclear receptor gene nur77 by nerve growth factor and membrane depolarization in PC12 cells. J. Biol. Chem. 268, 9148–55 (1993). 8473354

[b37] Enslen H. & Soderling T. R. Roles of calmodulin-dependent protein kinases and phosphatase in calcium-dependent transcription of immediate early genes. J. Biol. Chem. 269, 20872–7 (1994). 7520433

[b38] Fass D. M., Butler J. E. F. & Goodman R. H. Deacetylase activity is required for cAMP activation of a subset of CREB target genes. J. Biol. Chem. 278, 43014–9 (2003). 12939274 10.1074/jbc.M305905200

[b39] Tokuoka H., Hatanaka T., Metzger D. & Ichinose H. Nurr1 expression is regulated by voltage-dependent calcium channels and calcineurin in cultured hippocampal neurons. Neurosci. Lett. 559, 50–5 (2014). 24291696 10.1016/j.neulet.2013.11.033

[b40] Hanna R. N. *et al.* The transcription factor NR4A1 (Nur77) controls bone marrow differentiation and the survival of Ly6C- monocytes. Nat. Immunol. 12, 778–85 (2011). 21725321 10.1038/ni.2063 PMC3324395

[b41] Nahrendorf M. *et al.* The healing myocardium sequentially mobilizes two monocyte subsets with divergent and complementary functions. J. Exp. Med. 204, 3037–47 (2007). 18025128 10.1084/jem.20070885 PMC2118517

[b42] Osadchii O. E. Cardiac hypertrophy induced by sustained beta-adrenoreceptor activation: pathophysiological aspects. Heart Fail. Rev. 12, 66–86 (2007). 17387610 10.1007/s10741-007-9007-4

[b43] Den Haan A. D. *et al.* Organ explant culture of neonatal rat ventricles: a new model to study gene and cell therapy. PLoS One 8, e59290 (2013). 23516623 10.1371/journal.pone.0059290 PMC3596330

[b44] Ter Welle H. F., Baartscheer A., Fiolet J. W. & Schumacher C. A. The cytoplasmic free energy of ATP hydrolysis in isolated rod-shaped rat ventricular myocytes. J. Mol. Cell. Cardiol. 20, 435–41 (1988). 3210251 10.1016/s0022-2828(88)80135-4

[b45] Baartscheer A., Schumacher C. A., Opthof T. & Fiolet J. W. The origin of increased cytoplasmic calcium upon reversal of the Na+/Ca(2+)-exchanger in isolated rat ventricular myocytes. J. Mol. Cell. Cardiol. 28, 1963–73 (1996). 8899555 10.1006/jmcc.1996.0189

[b46] Shy D. *et al.* PDZ domain-binding motif regulates cardiomyocyte compartment-specific NaV1.5 channel expression and function. Circulation 130, 147–60 (2014). 24895455 10.1161/CIRCULATIONAHA.113.007852

[b47] Van Deel E. D. *et al.* Exercise training does not improve cardiac function in compensated or decompensated left ventricular hypertrophy induced by aortic stenosis. J. Mol. Cell. Cardiol. 50, 1017–25 (2011). 21291889 10.1016/j.yjmcc.2011.01.016

