# Supplementary material for: Orphan nuclear receptor Nur77 affects cardiomyocyte calcium homeostasis and adverse cardiac remodelling
Source: Sci Rep. 2015 Oct 21;5:15404. doi: 10.1038/srep15404 (PMC4613907; doi:10.1038/srep15404)
Supplement: Supplementary Information [file srep15404-s1.pdf]

SUPPLEMENTARY INFORMATION

Orphan nuclear receptor Nur77 affects cardiomyocyte calcium homeostasis and adverse cardiac remodelling.

Lejla Medzikovic[1,2], Cees A Schumacher[3], Arie O Verkerk[4], Elza D van Deel[5], Rianne Wolswinkel[3], Ingeborg van der Made[3], Natascha Bleeker[1], Daniella Cakici[1], Maarten MG van den Hoogenhof[3], Farid Meggouh[1], Esther E Creemers[3], Carol Ann Remme[3], Antonius Baartscheer[3], Robbert J de Winter[2], Carlie JM de Vries[1], E Karin Arkenbout[2,6], Vivian de Waard[1*]

[1]Department of Medical Biochemistry, [2]Department of Cardiology, [3]Department of Experimental Cardiology, [4]Department of Anatomy, Embryology and Physiology, Academic Medical Center, University of Amsterdam, The Netherlands

[5]Division of Cardiology, Thoraxcenter, Erasmus Medical Center, University of Rotterdam, The Netherlands

[6]Department of Cardiology, Tergooi Hospital, Blaricum, The Netherlands

CORRESPONDING AUTHOR:

V de Waard, PhD

Academic Medical Center Amsterdam, Department of Medical Biochemistry, K1-114

Meibergdreef 9, 1105 AZ Amsterdam, The Netherlands

Tel: + 31 20 5665129 / Fax: + 31 20 6915519 / E-mail: v.dewaard@amc.uva.nl

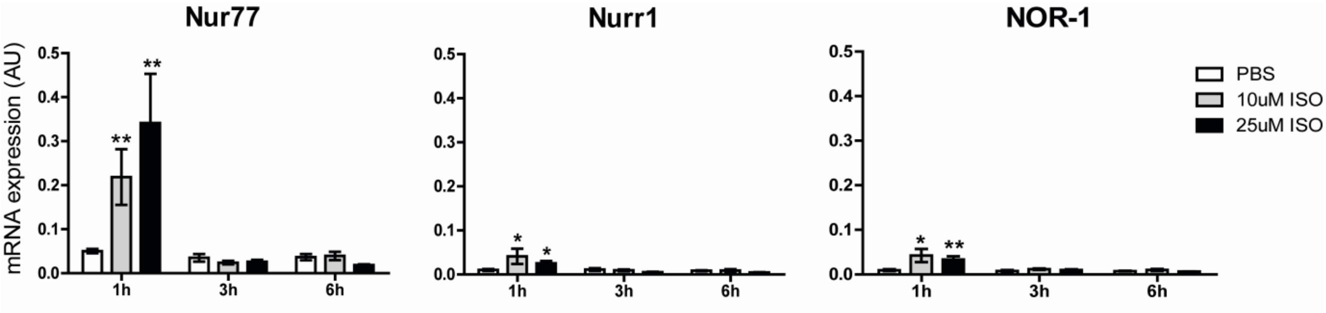

Supplementary Figure S1. NR4A gene expression in cardiomyocytes after isoproterenol stimulation. Expression of all three members of the NR4A subfamily of nuclear receptors is significantly up-regulated after 1h of isoproterenol stimulation, with Nur77 mRNA being most abundant. After 3 and 6h of stimulation, expression of all three NR4As is decreased to control levels. Data presented as median and minimum/maximum values.

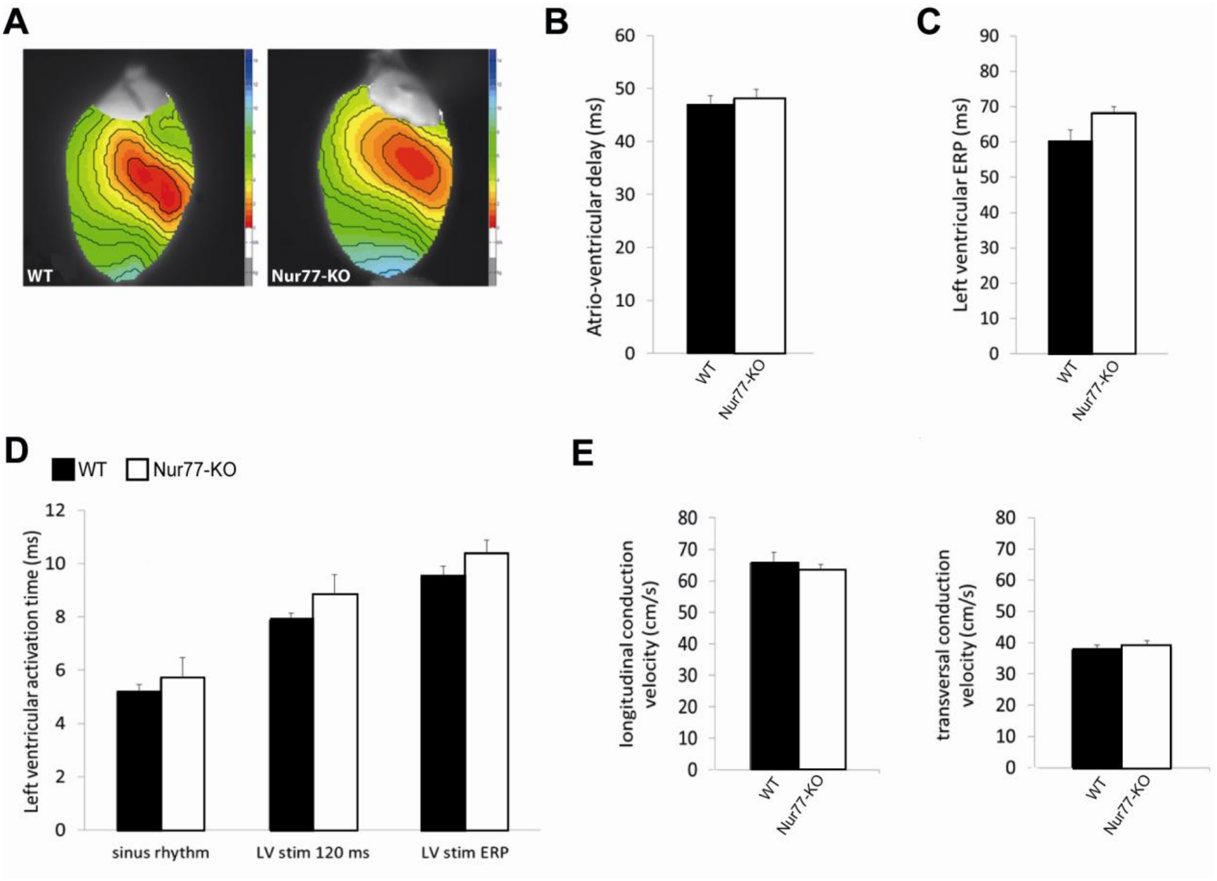

Supplementary Figure S2. Unaltered conduction in Nur77-KO hearts. Optical mapping experiments were performed in WT (n=8) and Nur77-KO (n=7) hearts. **A.** Typical examples of left ventricular (LV) activation maps of a WT and Nur77-KO heart during central LV stimulation at a cycle length of 120 ms. No differences were observed between WT and Nur77-KO in atrio-ventricular conduction delay during right atrial stimulation at 120 ms **(B)** LV effective refractory period (ERP; **C**; trend p=0.06), total LV activation times during sinus rhythm, LV central stimulation at 120 ms and during LV central stimulation at the ERP value **(D)** or LV longitudinal and transversal conduction velocities during LV central stimulation at 120 ms. Data presented as mean+SEM.

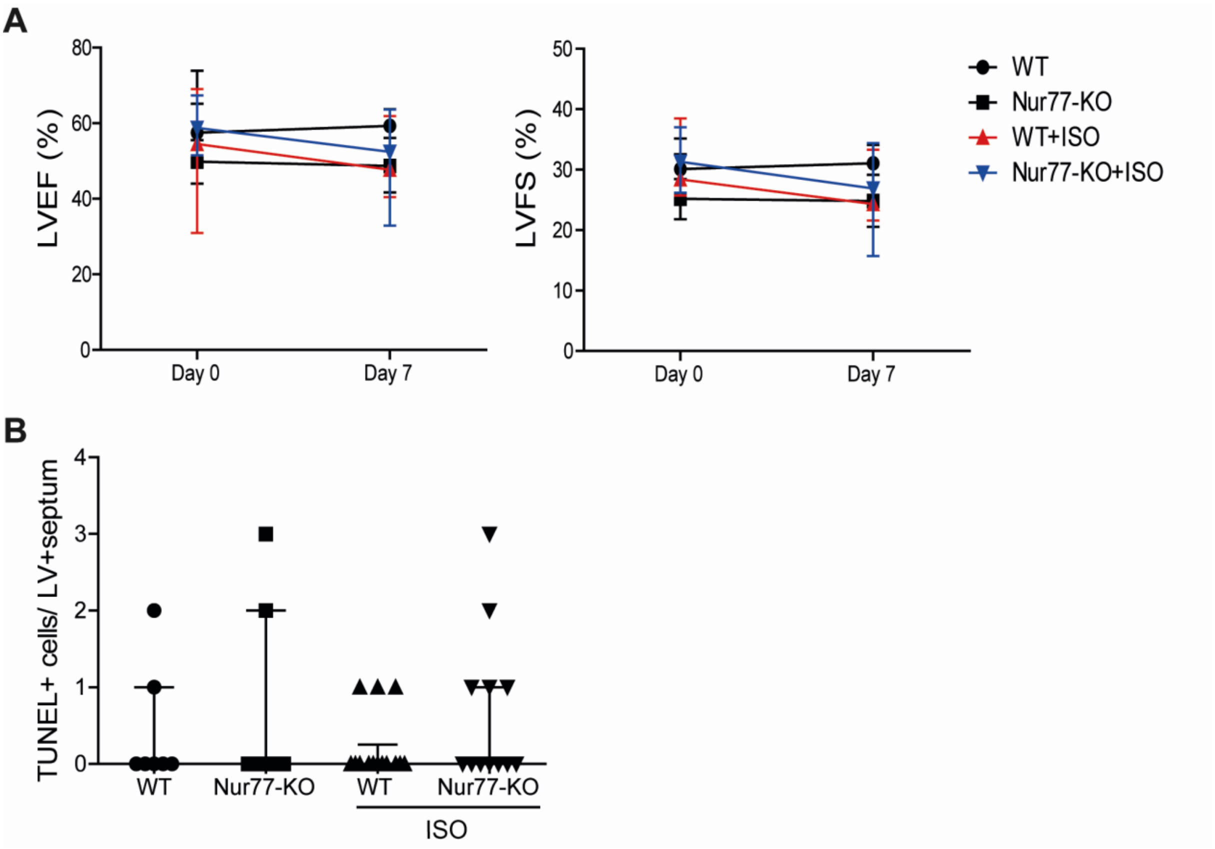

Supplementary Figure S3. Cardiac function and apoptosis after chronic isoproterenol stimulation. A. Cardiac function after 1 week of isoproterenol stimulation as assessed in all WT mice (n=16), the surviving 13 Nur77-KO mice and control groups (n=9 each). Although a hypertrophic response is established in both WT and Nur77-KO mice after 1 week of isoproterenol stimulation, no difference in left ventricular ejection fraction (LVEF) and fractional shortening (LVFS) was evident compared to baseline or between groups. **B.** Apoptotic cells as assessed by TUNEL staining. No significant difference between the numbers of apoptotic cells in left ventricles of WT and Nur77-KO mice after 1 week of isoproterenol stimulation was observed (median is 0 in all groups). Data presented as median and minimum/maximum values.

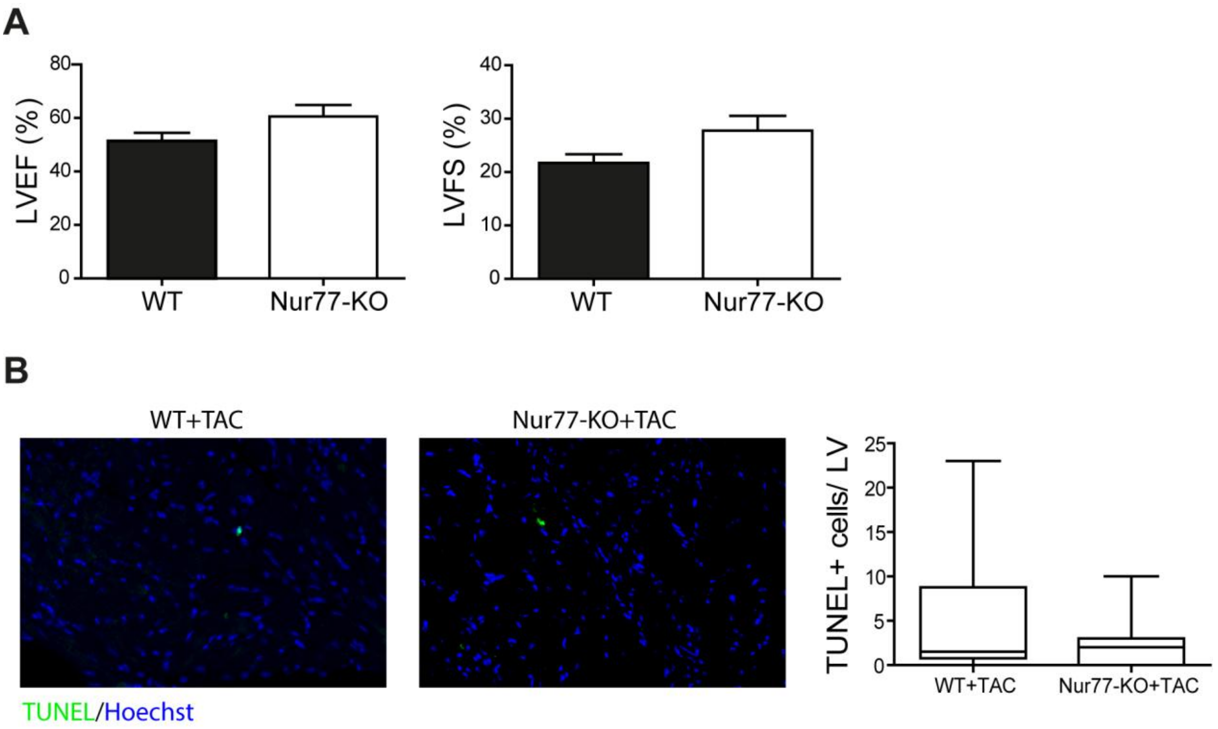

Supplementary Figure S4. Cardiac function and apoptosis after TAC. A. Cardiac function after 28 days of transverse aortic constriction. Left ventricular ejection fraction (LVEF) and fractional shortening (LVFS) were assessed in all WT (n=13) and Nur77-KO (n=11) mice. Nur77-KO mice exhibit a trend towards enhanced cardiac function. Data presented as mean+SEM. **B.** Apoptotic cells as assessed by TUNEL staining. No significant difference between the numbers of apoptotic cells in left ventricles of WT and Nur77-KO mice after 28 days of aortic constriction was observed. Data presented as median and minimum/maximum values.
